# Vorhofflimmern beim Sportler: Häufigkeit, Diagnose und Therapie

**DOI:** 10.1007/s00399-022-00913-4

**Published:** 2022-12-29

**Authors:** Agne Adukauskaite, Markus Stühlinger

**Affiliations:** 1 Univ. Klinik für Innere Medizin III, Kardiologie und Angiologie, Medizinische Universität Innsbruck, Tirol-Kliniken, Anichstraße 35, 6020 Innsbruck, Österreich; 2 Innsbruck, Österreich

**Keywords:** Rhythmusstörung, Athlet:innen, Sport, Katheterablation, Antiarrhythmika, Rhythm disturbances, Athletes, Sport, Catheter ablation, Anti-arrhythmic drugs

## Abstract

**Hintergrund:**

Bei Freizeit- und Spitzensportlern nimmt die Inzidenz von Vorhofflimmern (VHF) abhängig von der Intensität der sportlichen Belastung im Mittel um das 2,5-Fache zu. Die Festlegung einer genauen Dauer bzw. der Belastungsintensität, ab welcher das VHF-Risiko erhöht wird, ist allerdings schwierig. Die pathophysiologischen Mechanismen der Flimmerarrhythmie bei Athlet:innen setzen sich aus Pulmonalvenen-Ektopien als Trigger, myokardialen Veränderungen wie Fibrose und Remodeling-Prozessen und Modulatoren wie die Veränderungen des autonomen Nervensystems zusammen. Aber auch der gastroösophageale Reflux scheint eine wichtige Rolle zu spielen.

**Material und Methoden:**

Die Diagnose eines Vorhofflimmerns erfolgt klassischerweise mittels 12-Kanal- oder Holter-EKG, Arrhythmie-Aufzeichnungen auf Brustgurten und Pulsuhren sind für die Differenzierung der Arrhythmie nicht ausreichend. Wearables mit der Möglichkeit einer EKG-Aufzeichnung können aber ebenfalls zum Screening eingesetzt werden. Auf eine VHF-Dokumentation bei Sportler:innen sollte zunächst eine Trainingspause und eine genaue kardiologische auch mögliche nichtkardiale Diagnostik erfolgen. Danach ist die Evaluierung einer oralen Antikoagulation von Bedeutung. Antiarrhythmische Dauertherapien werden von Sportler:innen meist nicht toleriert oder gewünscht. Daher kommt als therapeutische Möglichkeiten meist nur eine *Pill-in-the-pocket*-Therapie mit einem Antiarrhythmikum oder aber eine Katheterablation in Frage.

## Abstract (en)

**Background:**

The incidence of atrial fibrillation (AF) is increased by an average of approximately 2.5-fold in recreational and elite athletes, depending on the intensity of exercise. It is, however, difficult to determine the exact duration or intensity of exercise that increases the risk of AF. The pathophysiological mechanisms of AF in athletes are a combination of pulmonary vein ectopy as a trigger, myocardial changes such as fibrosis and remodeling processes, and modulators such as changes in the autonomic nervous system. However, gastroesophageal reflux also seems to play an important role.

**Material and methods:**

The classic AF diagnosis is performed by means of 12-lead or Holter ECG; arrhythmia recordings via chest belts and pulse watches are not sufficient for the differentiation of the arrhythmia. However, wearables with the capability of ECG recording can also be used for AF screening. The first AF documentation in an athlete should be followed by cessation of physical exercise and initiation of detailed cardiac diagnostics. Thereafter, evaluation of oral anticoagulation is important. Long-term antiarrhythmic therapies are usually not tolerated or desired by athletes. Thus, valuable therapeutic options are either a “pill in the pocket” therapy with antiarrhythmic drugs or catheter ablation.

Vorhofflimmern (VHF) ist die am häufigsten auftretende anhaltende Herzrhythmusstörung des Menschen. Dabei wird die sehr hohe Herzfrequenz (ca. 300/min) in den Vorhöfen schnell und unregelmäßig ventrikulär übergeleitet. Die dadurch entstehende Arrhythmie ist mit einem erhöhten Risiko von Schlaganfällen verbunden und führt v. a. bei anhaltender Tachykardie zur Herzinsuffizienz [1]. Die Inzidenz von VHF steigt mit dem Alter von Patient:innen und mit assoziierten Komorbiditäten wie arterieller Hypertonie, Adipositas oder strukturellen Herzerkrankungen. Allerdings wurde auch im Rahmen hoher Ausdauerbelastungen bei gesunden Spitzensportler:innen ein deutlich erhöhtes Risiko für das Auftreten von VHF beobachtet [2].

## Häufigkeit

Die Ausübung von Ausdauersport hat grundsätzlich positive Auswirkungen auf die allgemeine körperliche Gesundheit. So stellt schon regelmäßige und moderate körperliche Ertüchtigung von mindestens 2 h pro Woche die wirkungsvollste Lebensstil-Intervention dar und kann das Auftreten von kardiovaskulären (Schlaganfall, arterielle Hypertonie und koronare Herzerkrankung), aber auch von onkologischen (z. B. Prostatakarzinome, Brustkrebs etc.) Erkrankungen signifikant verhindern [3]. Andererseits ist seit vielen Jahren bekannt, dass Spitzensportler:innen langfristig ein signifikant erhöhtes Risiko des Auftretens von VHF aufweisen: In der größten und rezentesten Metaanalyse [4] wurden insgesamt 13 Studien zu VHF mit fast 7000 Athlet:innen aus vielen verschiedenen Sportarten inkludiert und mit ca. 64.000 Kontrollpersonen verglichen. Die Gegenüberstellung der beiden Gruppen ergab, dass Athlet:innen verglichen mit der Kontrollgruppe ein ca. 2,5-fach erhöhtes Risiko der Flimmerarrhythmie aufwiesen, wobei Langläufer am wenigsten, aber jüngere Sportler:innen (< 55 Jahre) und Teilnehmer an gemischten Sportarten (z. B. Fußball, Rugby) besonders stark betroffen waren [4]. Aufgrund der schwierigen Vergleichbarkeit der verschiedenen Sportarten und der seltenen Fallzahlen ist die Festlegung einer genauen Dauer bzw. der Belastungsintensität, ab welcher das VHF-Risiko erhöht wird, schwierig. Auch kann auf Basis der bestehenden Daten nicht zwischen *gesunden* und *gefährlichen* Sportarten differenziert werden. Daher bleibt vorerst unklar, wo genau die Grenze zur ungesunden Sportausübung in Bezug auf die VHF-Inzidenz liegt [5].

## Mechanismen von VHF bei Sportler:innen

Mehrere pathophysiologische Mechanismen scheinen einen Einfluss auf die Entstehung von VHF bei Athlet:innen zu haben. Die Hypothesen zur Pathophysiologie dieser Rhythmusstörung basieren dabei in erster Linie auf Tierexperimenten, Bildgebungsstudien und elektrophysiologischen Untersuchungen [6].

### Trigger: Pulmonalvenen-Ektopien

Klar erscheint, dass die Arrhythmie wie auch bei Nichtsportler:innen durch schnelle meist aus den Pulmonalvenen stammende Ektopien ausgelöst wird [7]. Diese Trigger können durch den bei Sportler:innen häufig beobachteten Vagotonus begünstigt werden, aber auch leistungsfördernde Substanzen (Anabolika, Amphetamine) und bestimmte Nahrungsergänzungsmittel (z. B. Creatin) können zu verstärktem Auftreten von supraventrikulären Extrasystolen und die sukzessive Auslösung von VHF führen [8].

Andererseits wird postuliert, dass neben diesen Triggern gerade bei Sportler:innen spezielle Modulatoren und Substrat für das Auftreten und Persistieren von VHF beteiligt sind [6]. Zum pathophysiologischen Substrat werden anatomische, molekulare und histologische Veränderungen („*atriales Remodeling*“) wie Dilatation der Vorhöfe, Veränderungen der Ionenkanäle und Mechanorezeptoren, Entzündungsprozesse und interstitielle Fibrose gezählt. Als Modulatoren gelten die autonome Stimulation, gastroösophageale Refluxkrankheit sowie Elektrolytstörungen und Flüssigkeitsveränderungen.

### Substrat: kardiales Remodeling, Fibrose und Inflammation

Intensives Kraft- aber auch Ausdauertraining kann zunächst zu biatrialer und später zu ventrikulärer Dilatation (durch Volumen- und Druckbelastung) des Herzens führen [9]. Eine Reihe von Studien konnten zeigen, dass vor allem die Dilatation der Vorhöfe mit einer erhöhten Inzidenz VHF assoziiert ist [10].

Die Assoziation zwischen interstitieller myokardialer Fibrose und trainingsbedingtem VHF ergibt sich vorwiegend aus Tiermodellen. In diesen konnte Fibrose histologisch nachgewiesen und eine erhöhte Expression von Fibrosemarkern in Antwort auf verstärkte körperliche Aktivität beobachtet werden [11]. Darüber hinaus konnten Studien mit langjährigen Sportler:innen in fortgeschrittenem Lebensalter zeigen, dass gerade diese Population eine erhöhte Rate an biochemischen und bildgebenden Zeichen einer myokardialen Fibrose in der kardialen MRT aufwies [10].

Es wird angenommen, dass diese Fibrose die Konsequenz einer chronischen Inflammation, ausgelöst durch besonders starke körperlicher Anstrengung, ist. Insbesondere zeigten Athlet:innen nach starker körperlicher Ausdauerbelastung hohe Werte von proinflammatorischen Zytokinen im Blut zusammen mit echokardiographischen [12] und MR-tomographischen Hinweisen auf eine myokardiale Dysfunktion [13]. Die atriale Fibrose ist wiederum ein verlässlicher Marker für eine erhöhte Inzidenz von VHF.

### Modulatoren: Aktivierung des autonomen Nervensystems und gastroösophagealer Reflux

Ausdauersport führt zu Veränderungen des autonomen Nervensystems. Insbesondere sinkt bei Sportler:innen die Ruhe-Herzfrequenz, und es finden sich in elektrophysiologischen Messungen auch Hinweise für andere autonome Veränderungen mit Dominanz der vagalen Aktivierung [14]. VHF tritt bei jungen, gesunden Sportler:innen vorwiegend vagal getriggert auf und wird auf autonom bedingte Veränderungen der Leitungsgeschwindigkeit des Myokards und der atrialen Refraktärperiode zurückgeführt, welche die Entstehung von Makro-Reentry-Mechanismen begünstigt und sukzessive zu VHF führen kann [15].

Sportler:innen und auch nichtsportliche VHF-Patient:innen berichten häufig über Zusammenhänge von gastrointestinaler Symptomatik und der Initiierung der Flimmerarrhythmie. Tatsächlich steigert regelmäßiger Ausdauersport den gastroösophagealen Reflux [16] und erhöht die intraösophageale Azidität [17]. Darüber hinaus wiesen Marathonläufer:innen nach 20-km-Läufen vermehrte Zeichen einer Ösophagitis in der Endoskopie auf [18]. Eine große Observationsstudie mit 164.000 Patient:innen konnte darüber hinaus zeigen, dass eine gastroösophageale Refluxkrankheit die VHF-Inzidenz um fast 40 % erhöht [6].

Obwohl der Zusammenhang zwischen Sport, der gastroösophagealen Refluxerkrankung und VHF in mehreren Studien dargestellt werden konnte, gibt es derzeit noch keinerlei Evidenz, dass die Einnahme von Protonenpumpeninhibitoren oder abdominal-chirurgische Eingriffe die VHF-Neigung bei Athlet:innen verhindern können.

### Geschlecht

Der Genderfaktor ist hinsichtlich des VHF-Risikos unter Spitzensportler:innen aufgrund der deutlich geringeren Anzahl der in Studien inkludierten weiblichen Teilnehmern unklar [19]. Tatsächlich wurde der Zusammenhang zwischen VHF und Sport bei Athletinnen bisher noch nicht nachgewiesen [20]. Der protektive Einfluss des weiblichen Geschlechts auf die Entwicklung von Rhythmusstörungen dürfte an der Tatsache liegen, dass atriale Remodelingprozesse in Frauen geringer ausgeprägt sind und der Vagotonus bei weiblichen Sportlerinnen sowohl in Ruhe als auch während der körperlichen Aktivität dominiert [21].

## Diagnose

Der Goldstandard der Diagnose von VHF ist das 12-Kanal-EKG, in dem die Arrhythmie über einen Zeitraum von 10 s dokumentiert werden muss [1]. Über medizinische Betreuer und sportmedizinische Routine-Untersuchungen haben Sportler:innen oft einen leichten Zugang zu dieser essenziellen Diagnostik. Arrhythmien treten bei den betroffenen Personen aber auch sporadisch und nur sehr kurz auf, sodass zum Screening auf Herzrhythmusstörungen und zur Abklärung von Symptomen auf nichti-nvasive EKG-Monitore mit längerer Aufzeichnungsdauer zurückgegriffen wird [22]. Die Anwendung von Langzeit-EKGs ist allerdings durch moderaten Tragekomfort und Artefakte im Rahmen sportlicher Betätigung limitiert.

Daher werden gerade bei der Diagnostik von Arrhythmien bei Athleten oft Herzfrequenzmonitore und Pulsuhren verwendet. Bei diesen Methoden handelt es sich allerdings nicht um zertifizierte medizinische Geräte, weil diese im Vergleich zu Holter-EKGs durch ihre Artefaktanfälligkeit und die schlechte Differenzierung der Arrhythmien nur eine geringe Spezifität (55 %) und Sensitivität (15 %) für Herzrhythmusstörungen aufweisen [23]. Plethysmographische Apps auf Wearables und Brustgurtaufzeichnungen können daher auch in Athlet:innen zum Screening für Herzrhythmusstörungen und insbesondere VHF verwendet werden, für die Diagnose und der anschließenden Behandlung der Arrhythmie ist aber eine EKG-Dokumentation der Rhythmusstörung auf einem 12-Kanal-EKG oder über zumindest 30 s auf einem 1‑Kanal-Rhythmusstreifen notwendig [24].

Einige moderne Wearables sind in der Lage, beide diagnostischen Funktionen zu verbinden: So können diese Geräte Pulsunregelmäßigkeiten über eine plethysmographische Messung erkennen und den Träger im Anfall zur Aufzeichnung eines Smartphone-EKGs auffordern. Dieses wird dann elektronisch gespeichert und kann anschließend durch einen Fachmann entsprechend interpretiert werden. Tatsächlich konnten mit dieser Technologie in kleinen Fallserien auch bei Athleten eine Reihe von Tachykardien inklusive VHF dokumentiert und diagnostiziert werden [25, 26].

Im Anschluss an die Diagnose von VHF muss eine Differenzierung der Verlaufsform der Rhythmusstörung und eine kardiologische Abklärung durch einen Experten erfolgen. Bei neu diagnostiziertem und nur kurz aufgetretenem VHF ist eine sportmedizinische Untersuchung inklusive Labor und Echokardiographie sowie die Verhinderung möglicher Trigger (z. B. Dehydrierung, Elektrolytstörung) ausreichend. Im Falle von wiederkehrenden Episoden muss aber einerseits über eine Thromboembolieprophylaxe und andererseits eine Therapie zur Terminierung und Verhinderung von weiteren Episoden erwogen werden.

## Therapie

Die wichtigste Entscheidung in der Therapie von VHF ist die orale Antikoagulation (oAK). So kann die Einleitung einer entsprechenden gerinnungshemmenden Therapie das Risiko eines Hirninfarkts oder einer Thromboembolie um ca. 70 % reduzieren. Während eine Hemmung der Thrombozytenaggregation durch ASS oder P2Y12 Inhibitoren bei Patient:innen mit VHF heute keine Rolle mehr spielt, hat die Einführung der direkten Antikoagulanzien (Apixaban, Dabigatran, Edoxaban und Rivaroxaban) die Verwendung der oAK dramatisch vereinfacht. Dennoch erhöhen jegliche Antikoagulanzien auch das Blutungsrisiko, das selbstverständlich bei Teilnehmer:innen an Geschwindigkeits- oder Kontaktsportarten an sich schon erhöht ist [27]. Die Einleitung einer oAK muss daher abhängig vom thrombembolischen Risiko – gemessen durch den CHA_2_DS_2_-VASc Score – im Verhältnis zum Blutungsrisiko mit dem betroffenen Athleten/der betroffenen Athletin erfolgen [1]. Da die Spitzensportler:innen generell eine junge und sonst gesunde Population repräsentieren (CHA_2_DS_2_-VASc Score < 2), kann meist auf die oAK verzichtet werden.

Die Einleitung von pharmakologischen oder interventionellen Maßnahmen zur Kontrolle von VHF hängt in erster Linie von der Symptomatik und insbesondere der Leistungseinschränkung der Sportler:in durch die Arrhythmie ab. So hindert asymptomatisches VHF die Betroffenen nicht am Ausüben des Spitzensports, solange keine strukturelle Herzerkrankung und eine adäquate Herzfrequenzkontrolle vorliegt [28]. Durch den Wegfall der atrialen Kontraktion, der AV-Synchronität und der meist tachykarden ventrikulären Überleitung von VHF kommt es bei Auftreten der Rhythmusstörung während sportlicher Betätigung allerdings in den meisten Fällen zu einer ausgeprägten Symptomatik [29]. Daher werden von Athlet:innen mit VHF auch bei seltenen und selbstlimitierten Episoden der Arrhythmie frequenz- oder rhythmuskontrollierende Maßnahmen gefordert.

Eine Herzfrequenzkontrolle mittels Betablocker oder Kalziumantagonisten verbessert die Symptomatik bei Leistungssportler:innen nur moderat. Darüber hinaus reduzieren diese Medikamente über ihre negativ-chronotrope Wirkung auch die Leistungsfähigkeit während körperlicher Anstrengung und können die bei Athlet:innen häufig beobachteten nächtlichen Bradykardien verstärken. Letztlich ist die Einnahme von Betablockern bei manchen Sportarten auch durch Anti-Doping-Gesellschaften verboten. Insgesamt kann eine frequenzkontrollierende Therapie daher bei Spitzensportler:innen mit nicht permanentem VHF in den meisten Fällen nicht empfohlen werden [30].

Antiarrhythmika der Klasse I und III, wie Propafenon, Flecainid und Amiodaron, können Episoden von VHF effektiver verhindern als Betablocker. Die meisten dieser Substanzen (außer Flecainid) weisen jedoch ebenfalls eine herzfrequenzsenkende und damit leistungseinschränkende Wirkung auf. Darüber hinaus ist die Langzeiteinnahme von Antiarrhythmika mit einem hohen Risiko von Nebenwirkungen verbunden, und das proarrhythmogene Risiko von Klasse-IC-Antiarrhythmika ist mit steigender Herzfrequenz erhöht. Die am besten praktikable pharmakologische Maßnahme ist aus diesen Gründen die bedarfsweise Einnahme eines Klasse-IC-Antiarrhythmikums, als sog. *Pill-in-the-pocket*-Strategie zur Terminierung von intermittierendem VHF [31]. Wichtig erscheint in diesem Zusammenhang zu erwähnen, dass bei dieser Strategie der Rhythmisierung hohe Dosen der oralen Antiarrhythmika eingenommen und daher auch akute Bradykardien beobachtet werden. Aus diesem Grund sollte die Anwendung zunächst unter ärztlicher Aufsicht und in Ruhe erfolgen und ist auf sportliche Aktivität bis zum Abklingen der antiarrhythmischen Wirkung (3–4 h nach Einnahme) verzichtet werden [30].

**Figure 1:**
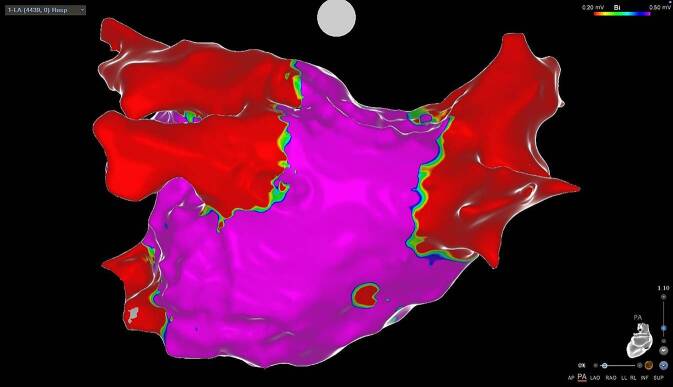

Die linksatriale Katheterablation mit Isolation der Pulmonalvenen-Ostien (PVI) hat sich in den letzten Jahren zu einem höchst effektiven interventionellen Verfahren zur Verhinderung von VHF entwickelt (Abb. 1). Der Eingriff selbst ist zwar komplex und auch mit Risiken verbunden, allerdings ist die PVI wirksamer in der Erhaltung des Sinusrhythmus als eine antiarrhythmische Therapie [32]. Da bei niedrigem CHA_2_DS_2_VASc-Score zwei Monate nach dem Eingriff meist auf die oAK verzichtet werden und schon nach einem Monat ohne Arrhythmie auch wieder mit sportlicher Betätigung begonnen werden kann [30], wird diese Therapieform von den betroffenen Sportler:innen oft bevorzugt. Bis dato konnte die Wirksamkeit und Sicherheit der PVI bei Athlet:innen im Vergleich zur rein medikamentösen Therapie nicht in randomisierten Studien bewiesen werden. Kleinere Beobachtungsstudien konnten allerdings zeigen, dass die Effektivität (d. h. die langfristige Erhaltung des Sinusrhythmus) der Katheterablation bei Ausdauersportler:innen mit der bei Patient:innen ohne strukturelle Herzerkrankung vergleichbar sind [33, 34]. Auch erreichen fast 80 % aller Athlet:innen nach erfolgreicher Katheterablation ihre ursprüngliche Leistungsfähigkeit wieder. Einschränkend wurde aber beobachtet, dass gerade Spitzensportler:innen im Langzeit-Follow-up (2 Jahre nach Ablation) häufiger ein VHF-Rezidiv oder atypisches Vorhofflattern als Konsequenz der linksatrialen Ablation erlitten [35].

Dieser Umstand weist auf die Tatsache hin, dass Leistungssport neben Ektopie-Triggern aus den Pulmonalvenen über o. g. Prozesse auch das atriale Remodeling mit elektrischen und strukturellen Veränderungen vorantreiben kann. Diese Prozesse können bislang noch nicht vollkommen pathophysiologisch erklärt und mit den derzeitigen Ablationsverfahren auch nicht therapiert werden. Eine Sportpause bis zur Ausheilung einer evtl. kardialen Grunderkrankung (z. B. Myokarditis), einer Katheterablation oder eine Reduktion des Trainingsprogramms (für ca. 3–4 Wochen) kann allerdings zu signifikanten Verbesserungen führen: So führte die Diagnose und Behandlung der Arrhythmie und die Reduktion des Trainingsausmaßes in einer großen Studie mit 1772 Spitzensportler:innen und einem langen Follow-up über 62 Monate zur Verbesserung der Symptomatik und Abnahme der VHF-Episoden, und der Leistungssport konnte nach einem beschwerdefreien Intervall (ca. 3–4 Wochen) wieder aufgenommen werden [36].

Insgesamt sollte aufgrund der vorliegenden Studiendaten bei Auftreten von VHF bei Leistungssportler:innen zunächst eine genaue kardiologische Abklärung zum Ausschluss einer strukturellen Herzerkrankung oder einer nichtkardialen Ursache erfolgen. Die Athlet:in sollte bis zur vollständigen Abklärung und Besserung der Symptomatik zu einer Trainingspause angehalten werden [30]. Bei Wiederbeginn der Arrhythmie kommt bei seltenen Episoden am ehesten eine bedarfsweise eingenommene Therapie eines Klasse-1C-Antiarrhythmikums in Frage. Bei Häufung der Episoden sollte dem/der Betroffenen eine linksatriale Katheterablation mit PVI angeboten werden.

## Fazit für die Praxis

- Ausdauersportarten führen zu einem bis zu 5‑fach erhöhten Risiko des Auftretens von Vorhofflimmern (VHF).
- Obwohl die genauen pathophysiologischen Mechanismen der VHF-Entwicklung unter Spitzensportler:innen noch unklar sind, geht man von einer besonderen Rolle einer autonomen (vorwiegend vagalen) Aktivierung und von einer chronischen Entzündungsreaktion durch Sport aus.
- Das Ausmaß und die Intensität von Sport (*Sportdosis*), ab welcher eine erhöhte Inzidenz von VHF auftritt, kann derzeit nicht sicher festgelegt werden.
- Für die Diagnose von VHF ist eine Aufzeichnung der Arrhythmie über 10 s in einem 12-Kanal- oder über 30 s in einem 1‑ oder 2‑Kanal-EKG notwendig.
- Als Therapie ist bei seltenen Episoden eine *Pill-in-the-pocket-*Strategie mit Klasse-1C-Antiarrhythmika (Flecainid, Propafenon) oder bei Häufung der Arrhythmie eine linksatriale Katheterablation zu empfehlen.
- Bis zum sicheren Ausschluss einer strukturellen Herzerkrankung und zur Besserung der Symptomatik ist eine Reduktion des Trainings und des Leistungssports ratsam.
